# Integrated analysis of high-throughput sequencing data reveals the key role of LINC00467 in the invasion and metastasis of testicular germ cell tumors

**DOI:** 10.1038/s41420-021-00588-9

**Published:** 2021-08-06

**Authors:** Hao Bo, Fang Zhu, Zhizhong Liu, Qi Deng, Guangmin Liu, Ruixue Li, Wenbing Zhu, Yueqiu Tan, Gang Liu, Jingyu Fan, Liqing Fan

**Affiliations:** 1 NHC Key Laboratory of Human Stem Cell and Reproductive Engineering, Institute of Reproductive and Stem Cell Engineering, School of Basic Medical Science, Central South University, Changsha, Hunan China; 2 Clinical Research Center for Reproduction and Genetics in Hunan Province, Reproductive and Genetic Hospital of CITIC-Xiangya, Changsha, Hunan China; 3 Hunan Cancer Hospital, Department of Urology, The Affiliated Cancer Hospital of Xiangya School of Medicine of Central South University, Changsha, Hunan China; 4 Department of Chemistry and Biochemistry, University of South Carolina, Orangeburg, SC USA

**Keywords:** Testicular cancer, Tumour biomarkers

## Abstract

Long noncoding RNAs (lncRNAs) are involved in various physiological and pathological processes. However, the role of lncRNAs in testicular germ cell tumor (TGCT) has been rarely reported. Our purpose is to comprehensively survey the expression and function of lncRNAs in TGCT. In this study, we used RNA sequencing to construct the lncRNA expression profiles of 13 TGCT tissues and 4 paraneoplastic tissues to explore the function of lncRNAs in TGCT. The bioinformatics analysis showed that many lncRNAs are differentially expressed in TGCT. GO and KEGG enrichment analyses revealed that the differentially expressed lncRNAs participated in various biological processes associated with tumorigenesis in *cis* and *trans* manners. Further, we found that the expression of LINC00467 was positively correlated with the poor prognosis and pathological grade of TGCT using WGCNA analysis and GEPIA database data mining. In vitro experiments revealed that LNC00467 could promote the migration and invasion of TGCT cells by regulating the expression of AKT3 and influencing total AKT phosphorylation. Further analysis of TCGA data revealed that the expression was negatively correlated with the infiltration of immune cells and the response to PD1 immunotherapy. In summary, this study is the first to construct the expression profile of lncRNAs in TGCT. It is also the first study to identify the metastasis-promoting role of LNC00467, which can be used as a potential predictor of TGCT prognosis and immunotherapeutic response to provide a clinical reference for the treatment and diagnosis of TGCT metastasis.

## Introduction

TGCTs are a large group of tumors that occur in the germinal epithelium of seminiferous tubules and can be categorized as seminoma (SEM) and non-seminoma (non-SEM); these tumors account for ~1% of all solid tumors in males and 98% of all testicular tumors [1]. The incidence of TGCT is exceptionally high in young men aged 20–40 years [2]. Data show that during recent years the incidence of TGCT has increased worldwide, resulting in an incidence rate of about 1 in 100,000 in China [3]. The primary clinical treatment method for TGCT is orchiectomy, supplemented with chemotherapy, and radiation therapy. Simultaneously, postoperative radiotherapy and chemotherapy can also adversely affect spermatogenesis and sexual function, and in some cases, cause secondary tumors and cardiovascular disease [4]. According to the European Guidelines for Urological Testicular Cancer, 15–20% of patients with stage I Seminoma, and ~30% of patients with non-seminoma develop recurrent metastases even after orchiectomy [4]. In summary, it is of great significance to explain the pathogenesis and molecular events that occur during the development, to identify more noninvasive and effective biomarker molecules and therapeutic targets, and to develop precision therapy options.

LncRNAs can regulate target gene expression at multiple levels, including epigenetic level, transcriptional level, post-transcriptional level, translational level, and post-translational level, as decoys, signals, scaffolds, or the induction of functional proteins, and participate in malignant tumor initiation or progression [5]. For example, AFAP1-AS1 affects AFAP1 protein levels and can promote cell metastasis by modulating actin filament integrity associated with nasopharyngeal carcinoma metastasis and poor prognosis [6]. LINC00152 is a lncRNA downstream of YAP1 that competes with miR-632 and miR-185-3p as an endogenous RNA that regulates the expression of FSCN1 to promote malignant proliferation and metastasis of colorectal cancer cells [7]. Previously, we found that LINC00467 could bind to AZGP1 and promote its degradation, activating the Akt signaling pathway to promote non-small cell lung cancer progression [8]. However, lncRNAs in TGCT have only rarely been investigated.

In this study, we performed high-throughput transcriptome sequencing to investigate the expression patterns, specific functions, and mechanisms of action of the lncRNAs in TGCT. We then performed a WGCNA analysis to select the lncRNA (LNC00467) that was most relevant to tumor metastasis and prognosis to be used in subsequent functional analysis and mechanism of action studies. We aimed to elucidate TGCT genome function from a perspective other than that of protein-coding genes, which will potentially provide novel molecular markers and therapeutic targets for TGCT diagnosis and treatment.

## Results

### Characterization of lncRNAs in TGCT tissues and paracancerous tissues

**Fig. 1:**
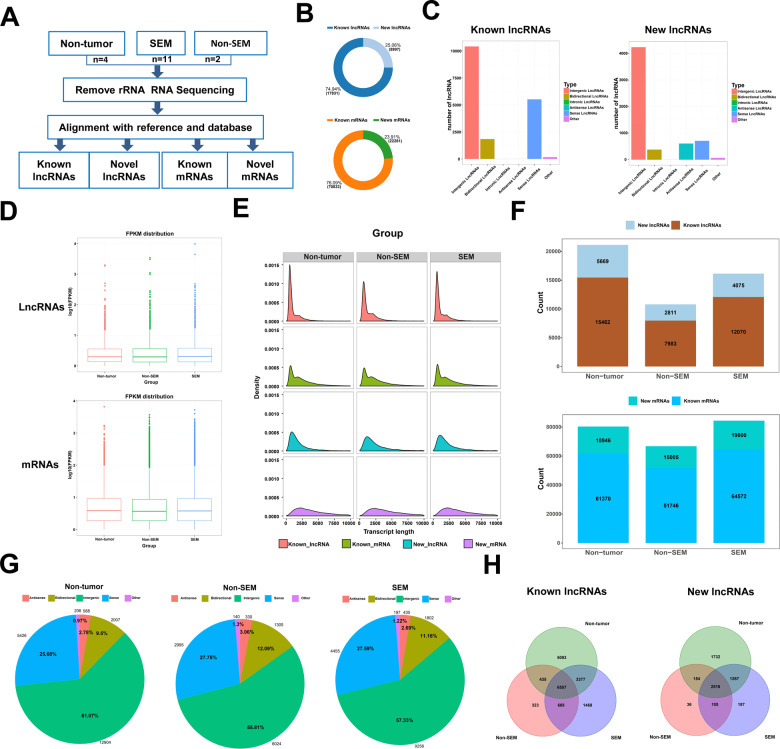
Overview of lncRNA Expression in TGCT Samples. **A** RNA sequencing flowchart. **B** Abundance of known lncRNAs to novel lncRNAs (top) and the ratio of known mRNAs to novel mRNAs (bottom). **C** Type abundance diagrams of known (left) and novel (right) lncRNAs. **D** Box plots of transcript expression levels in each group. **E** Length distribution density diagrams of known mRNAs and new mRNAs (pink and blue) and known lncRNAs and new lncRNAs (green and purple). **F** Distribution of the number of known lncRNAs and novel lncRNAs (top) and the number of known mRNAs and novel mRNAs (bottom) in each group. **G** Pie charts of the distribution of lncRNA types in the different groups of samples. **H** Wayne diagrams of known lncRNAs (left) and new lncRNAs (right) in each group.

The testicular tumors and paraneoplastic tissue samples were used for RNA sequencing (Fig. 1A). We found 17,931 known and 5,997 new lncRNA transcripts, as well as 70,833 known and 22,261 new mRNA transcripts in the samples (Fig. 1B). Further analysis of both known and new lncRNA transcripts revealed large differences between them, with intergenic lncRNAs being the most abundant in both groups, while intronic lncRNAs were the least abundant in both groups, while antisense lncRNAs were more abundant among the new lncRNA transcripts. Sense lncRNAs were the most abundant among the known lncRNA transcripts (Fig. 1C). After combining the known and new RNA transcripts, we found that the abundance of lncRNAs and mRNAs expression was relatively similar between the groups (Fig. 1D), with no significant differences.

In addition, we found that a transcript length of 0–2,500 nt was more common among known lncRNAs than new lncRNAs. However, there was no significant difference in the number of transcripts of known and novel mRNAs at this length range (Fig. 1E). Furthermore, we counted the number of lncRNA and mRNA transcripts in each group.

We found that a total of 21,131 lncRNA transcripts (known lncRNAs: 15,462, new lncRNAs: 5,669) were detected in the non-tumor group. In comparison, only 10,794 lncRNA transcripts (known lncRNAs: 7,983, new lncRNAs: 2,811) were detected in the non-SEM group, while 16,145 lncRNAs transcripts (known lncRNAs: 12,070, new lncRNAs: 4,075) were detected in the SEM. Overall, significantly fewer lncRNAs were detected in the SEM and non-SEM groups than in the non-tumor group. However, there was no significant difference in all three groups. The number of mRNAs in the SEM and non-tumor groups were similar, whereas the number of mRNAs detected in the non-SEM group was slightly less than that in the non-tumor group (Fig. 1F). Analysis of the different types of lncRNAs among the different sample groups revealed that the distribution of lncRNAs was similar. The proportion of each type of lncRNA was not significantly different (Fig. 1G). We also found that the lncRNAs expressed in each group showed both a high degree of similarity and specificity. There were 6,557 known lncRNA transcripts and 2,516 new lncRNA transcripts that were common to all three groups, while certain known and new lncRNA transcripts were specific to each group (Fig. 1H). These results indicate that multiple lncRNA transcripts are involved in the development of the different pathological types of testicular tissues.

### LncRNAs are differentially expressed in TGCT and paracancerous tissues

**Fig. 2:**
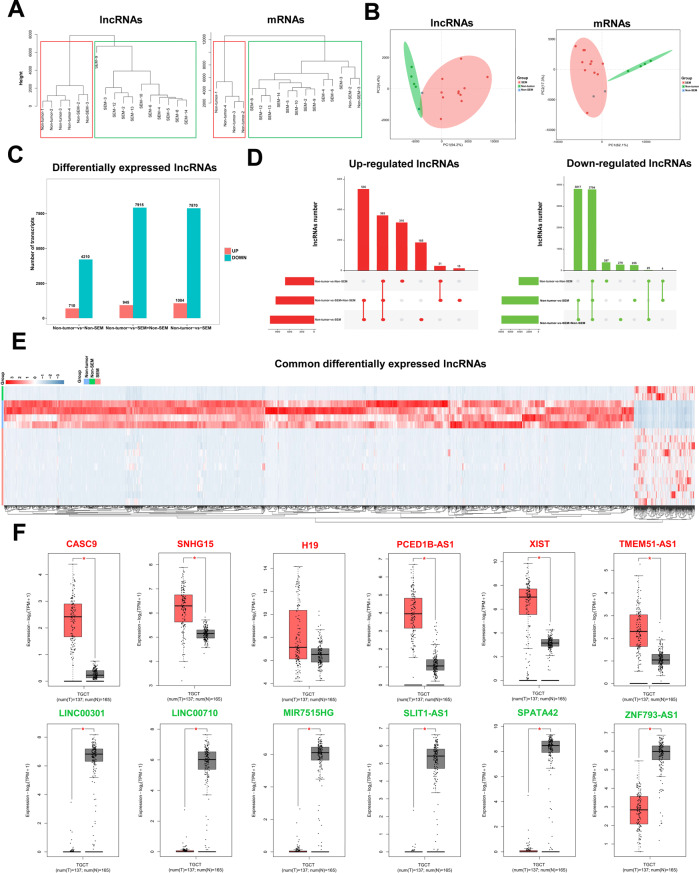
LncRNAs are significantly differentially expressed in TGCT. **A** Hierarchical clustering plots based on sample lncRNA expression (left) and sample mRNA expression (right). **B** PCA analysis plots based on sample lncRNA expression (left) and sample mRNA expression (right). **C** Histogram of DElncRNAs number between groups. **D** Upset plots of the distribution of upregulated lncRNAs (left) and downregulated lncRNAs (right) in each group. **E** Heatmap showed the common differentially expressed lncRNAs of seminoma and non-seminoma compared with adjacent tissues. **F** Verification of the common DElncRNAs in GEPIA database.

Hierarchical clustering analysis revealed that there were significant differences between the SEM and non-SEM groups. However, the results of hierarchical clustering analysis show that SEM and non-SEM are clustered in the same group (Fig. 2A). PCA analysis also yielded similar results (Fig. 2B).

Next, we identified the differentially expressed lncRNAs (DElncRNAs) between groups using edgeR package. We found a total of 710 significantly upregulated lncRNAs and 4,210 significantly downregulated lncRNAs in the non-SEM group, compared with the non-tumor group (Fig. 2C and Supplementary Fig. 1A). A total of 1,084 upregulated lncRNAs and 7,870 downregulated lncRNAs were significantly expressed in the SEM group, compared with the non-tumor group (Fig. 2C and Supplementary Fig. 1B). A total of 945 upregulated lncRNAs and 7,515 downregulated lncRNAs were significantly expressed in both the non-SEM+SEM groups, compared with the non-tumor group (Fig. 2C and Supplementary Fig. 1C). Further analysis of the DElncRNAs among the groups revealed that 363 lncRNAs were commonly upregulated and 3,794 lncRNAs were commonly downregulated in the above three comparison groups (Fig. 2D). The results mentioned above show that there were fewer upregulated lncRNAs in TGCT than downregulated lncRNAs. However, many differentially expressed lncRNAs were common to all pathological types. Significant differences in the expression patterns of the lncRNAs can be seen in all three groups (Fig. 2E). Subsequently, we randomly selected six significantly upregulated lncRNAs and six significantly downregulated lncRNAs from the common DElncRNAs for verification. Based on the TGCT data from GEPIA database, we found that all six upregulated lncRNAs were also upregulated in the GEPIA database. However, the difference for lncRNA H19 was not adequately significant (*P* value >0.01). The other six significantly downregulated lncRNAs were also significantly downregulated in the database, consistent with our results (Fig. 2F).

### LncRNAs are involved in the development of TGCT through multiple manners

**Fig. 3:**
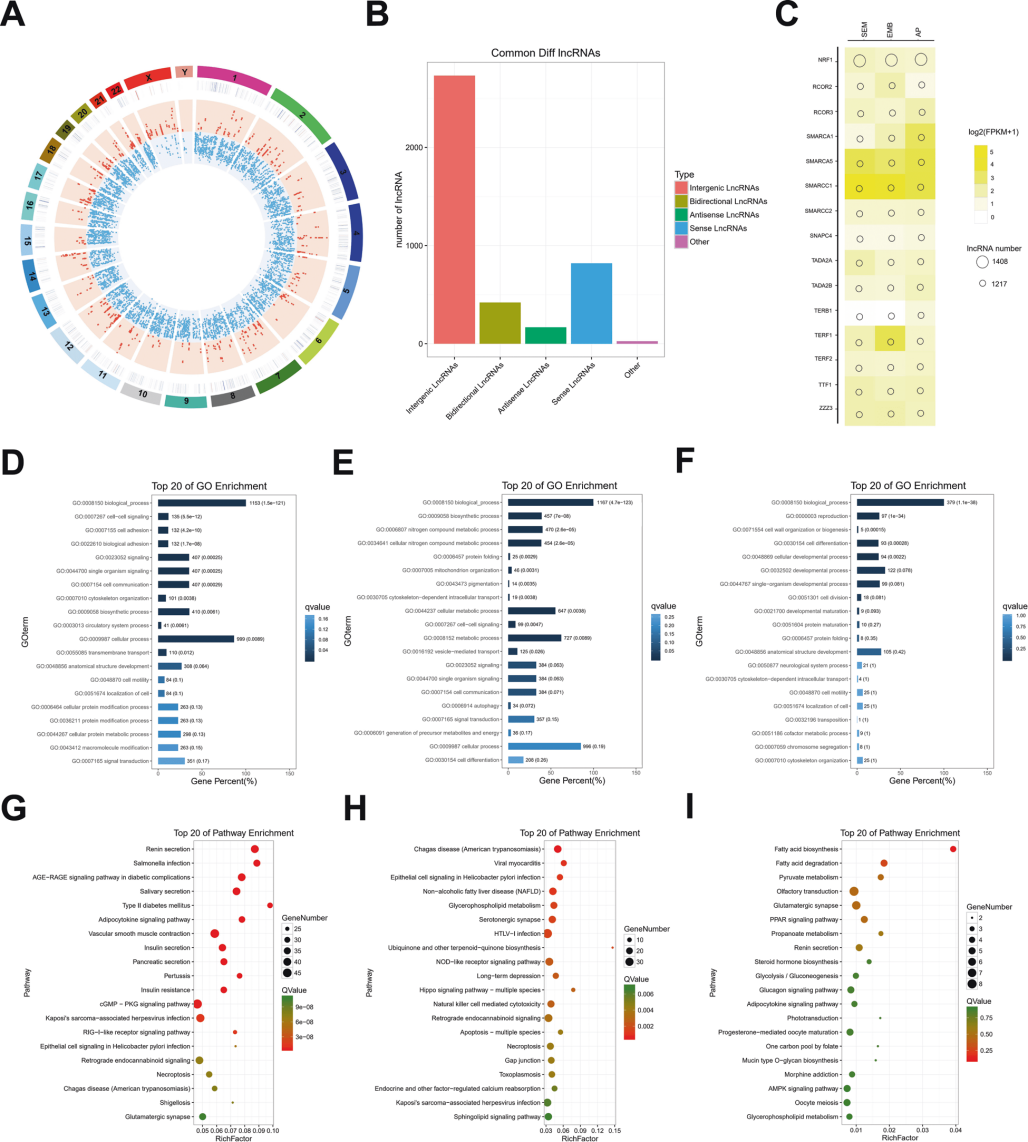
Functional enrichment analysis of the common DElncRNAs. **A** Circos plots show the distribution of differentially expressed lncRNAs on each chromosome. Upregulated lncRNAs are shown in red, while downregulated lncRNAs are shown in blue. **B** Bubble plots of the transcription factor enrichment analysis of co-differentially expressed lncRNAs. The darker the yellow color, the higher the transcription factor expression, while the bubble size indicates the number of lncRNAs. **C** Histogram of type distribution of common differentially expressed lncRNAs. **D**, **G** Plots showing the results of the GO and KEGG enrichment of the antisense lncRNA target genes. **E**, **H** Plots showing the results of the GO and KEGG enrichment of the lncRNA *cis*-action target genes. **F**, **I** Plots showing the results of the GO and KEGG enrichment of the lncRNA *trans*-action target genes.

The analysis of the distribution of common DElncRNAs in different groups showed that the DElncRNAs were primarily located on autosomal chromosomes and less frequently on X and Y chromosomes (Fig. 3A). And the DElncRNAs could be divided into four groups: intergenic lncRNAs, bidirectional lncRNAs, antisense lncRNAs, and sense lncRNAs (Fig. 3B). The transcription factors enrichment analysis of the DElncRNAs revealed that these lncRNAs might be regulated by multiple transcription factors, including the transcription factors SMARCA5 and SMARCC1, which were highly expressed in all three groups (Fig. 3C).

Many antisense lncRNAs regulate gene silencing, transcription, and mRNA stability by binding to the sense chain mRNAs. Therefore, we used R2NAplex software [9] to predict the complementary binding between antisense lncRNAs and mRNAs among these common DElncRNAs. Then, we conducted GO and KEGG enrichment analyses on the predicted target mRNAs. The results of the GO analysis mainly focused on the functional categories of “cell adhesion,” and “cytoskeleton organization” (Fig. 3D). The KEGG signaling pathway enrichment analysis revealed that these genes were mainly enriched in the “cGMP-PKG signaling pathway,” and “RIG-I-like receptor” (Fig. 3G).

The functions of lncRNAs are also associated with their neighboring protein-coding genes, and lncRNAs located upstream of genes may intersect promoters or other *cis*-acting elements of co-expressed genes, allowing for gene expression to be regulated at the transcriptional or post-transcriptional level. Therefore, we analyzed the *cis*-acting target genes of the above DElncRNAs. GO and KEGG enrichment analyses were performed on these target genes. The results of the GO enrichment analysis showed that the *cis*-acting target genes were mainly involved in “cytoskeleton-dependent intracellular transport,” and “cellular metabolic” (Fig. 3E). The results of the KEGG enrichment analysis showed that signaling pathways such as “nod-like receptor signaling pathway,” and “natural killer cell-mediated cytotoxicity” were significantly enriched (Fig. 3H).

Based on the basic principle of *trans*-acting target gene prediction, the function of the lncRNAs was not associated with the position of the coding genes. However, they were associated with the protein-coding gene co-expression. We used the Pearson correlation coefficient method to analyze the correlation between the expression of the lncRNAs and protein-coding genes among the samples to predict their target genes. The protein-coding genes with an absolute correlation value greater than 0.9 were included in the GO functional enrichment and KEGG pathway enrichment analyses, which were conducted to predict the main functions of the lncRNAs. The results of the GO enrichment analysis showed that these trans-acting target genes were mainly concentrated in the functional categories of “cell differentiation,” “cell developmental process,” and “cell motility” (Fig. 3F). The results of the KEGG pathway enrichment analysis showed that the target genes were mainly enriched in the “adipocytokine signaling pathway,” “Oocyte meiosis,” and other signaling pathways (Fig. 3I). These results suggest that lncRNAs are involved in the development of TGCT through different manners and different signaling pathways.

### Phenotype-associated lncRNA screening

**Fig. 4:**
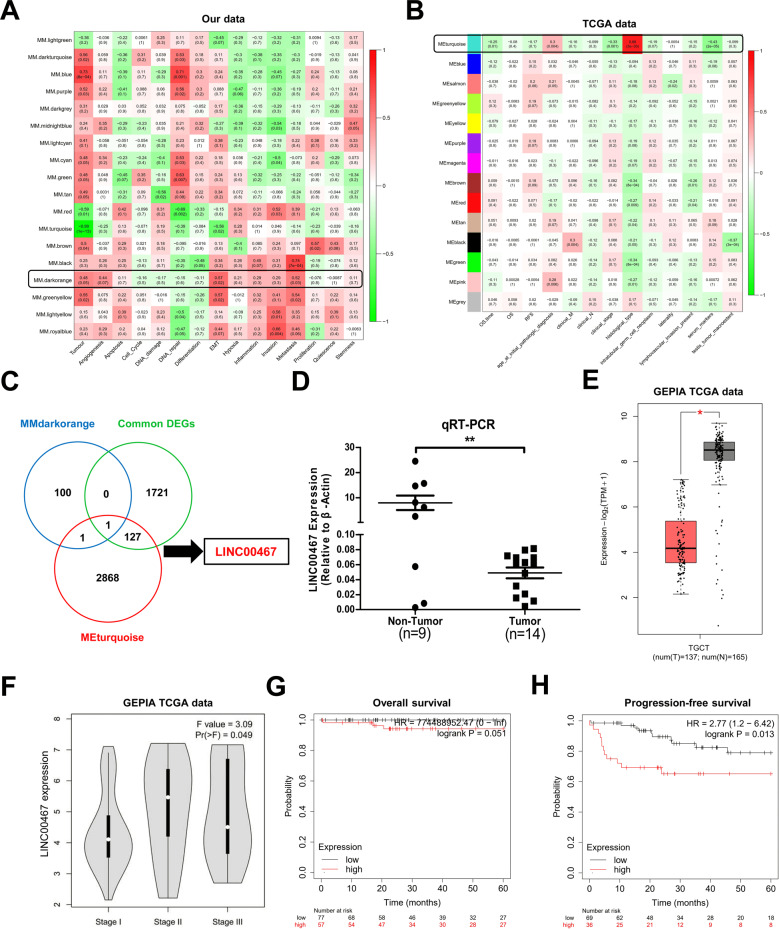
LINC00467 expression is significantly correlated with clinicopathological features, metastasis, and the prognosis of TGCT. **A**, **B** WGCNA analysis of the clinical phenotype module correlation using self-tested RNA sequencing data (**A**) and TGCT data from TCGA database (**B**). **C** MMdarkGreen module, Meturquoise module, and Wayne diagram of the differential lncRNAs. **D**, **E** Quantitative real-time PCR (**D**) and GEPIA database TCGA data (**E**) were used to verify the differential expression of LNC00467 in TGCT. **F** Expression level of LINC00467 in the TGCT dataset of the GEPIA database was associated with the pathological grade of TGCT. **G**, **H**, TGCT data from the Kaplan-Meier Plotter database were used to analyze the correlation between LINC00467 and the prognosis of TGCT patients.

To identify the modules and key lncRNAs most relevant to the clinical phenotypes of the TGCT, we first conducted a WGCNA analysis on our data. The module, MM.darkorange (Fig. 4A), was correlated with the clinical phenotypes of TGCT tumorigenesis, angiogenesis, EMT, and metastasis. Meanwhile, we also performed WGCNA analysis on TGCT data from TCGA. By calculating the correlation between the values of each module feature and the clinical phenotype, we found that the module, Meturquoise (Fig. 4B), was correlated with clinical phenotypes, such as overall patient survival time (OS time) and patient histological type. Next, we intersected lncRNAs of the MMdarkorange and Meturquoise modules with the common DElncRNAs obtained from the previous analysis to identify lncRNAs that were most relevant to TGCT generation and metastasis. As a result only one lncRNA, LNC00467, was intercepted (Fig. 4C). The differential expression of LINC00467 was also confirmed by qRT-PCR (Fig. 4D) and GEPIA data mining (Fig. 4E). Furthermore, we found that LINC00467 expression levels in tumor tissues of stage II and stage III TGCT patients were significantly higher than that of stage I TGCT patients (Fig. 4F). In addition, the analysis of the correlation between LINC00467 and patient prognosis showed that patients with a high level of LINC00467 expression in tumor tissues had a significantly lower 5-year overall survival and 5-year disease-free survival than patients with low levels of LINC00467 expression (Fig. 4G, H).

### Effects of LINC00467 on the migration, invasion, and clone formation of TGCT cells

**Fig. 5:**
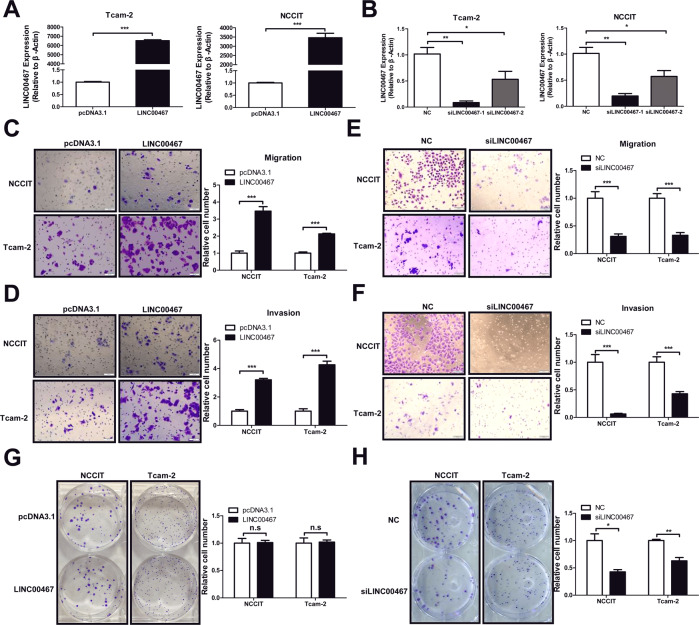
LINC00467 promotes TGCT cell migration and invasion. **A** qRT-PCR assay was used to detect LINC00467 overexpression in the cell lines. **B** qRT-PCR assay was used to detect the silencing efficiency of LINC00467 siRNA in the cell lines. The relative expression of LINC00467 in each group was calculated using β-actin as an internal reference and normalized to 1 in the NC siRNA group. **C**, **E** Transwell cell migration assays were used to determine the effects of LINC00467 overexpression (**C**) and the silencing of LNC00467 (**E**) on the migration ability of TGCT cells. **D**, **F** Transwell cell invasion assays were used to determine the effects of LINC00467 overexpression (**D**) and the silencing of LNC00467 (**F**) on the migration ability of TGCT cells. **G**, **H** Clone formation assay to detect the effect of LINC00467 overexpression (**G**) and the silencing of LNC00467 (**H**) on the colony-formation ability of TGCT cells. *P < 0.05; **P < 0.01; ***P < 0.001; n.s. no significance. All experiments were performed in triplicate. Scale bar = 100 μm.

Since TGCT can be divided into two types: seminoma and non-seminoma, we selected two cell lines Tcam-2 (seminoma cell line) [10] and NCCIT (non-seminoma cell line) [11] for further in vitro experiments. The expression levels of LINC00467 in NCCIT and Tcam-2 were determined using qRT-PCR to verify the effects of the overexpression and silencing of LINC00467 (Fig. 5A, B), followed by Transwell cell migration experiments. The results showed that LINC00467 overexpression significantly increased the number of cells that had migrated (Fig. 5C), while LINC00467 silencing significantly decreased the number of cells that had migrated (Fig. 5E). To investigate whether LINC00467 altered the invasive abilities of TGCT cells, we silenced LNC00467 or overexpressed LNC00467 in these two TGCT cell lines. The cells were then inoculated separately into a Matrigel-coated Transwell chamber, and their invasive capacity was assayed. LINC00467 overexpression significantly increased the number of tumor cells that invaded the subsurface of the Transwell chamber (Fig. 5D). In contrast, LINC00467 silencing significantly decreased the number of tumor cells that invaded the subsurface of the Transwell chamber (Fig. 5F).

More extensive metastases result from the gradual growth of a small number of cells that have colonized the tumor. To investigate whether LINC00467 is involved in this process, colony-formation assays were performed after the silencing or overexpression of LINC00467 in cells. We found that LINC00467 silencing significantly decreased the number of clones formed (Fig. 5H). However, the clone-forming ability of these cells was not significantly altered by LINC00467 overexpression (Fig. 5G), suggesting that LINC00467 expression is redundant in the clone-forming phenotype.

### LINC00467 participates in cell migration and invasion by regulating the transcription of AKT3

**Fig. 6:**
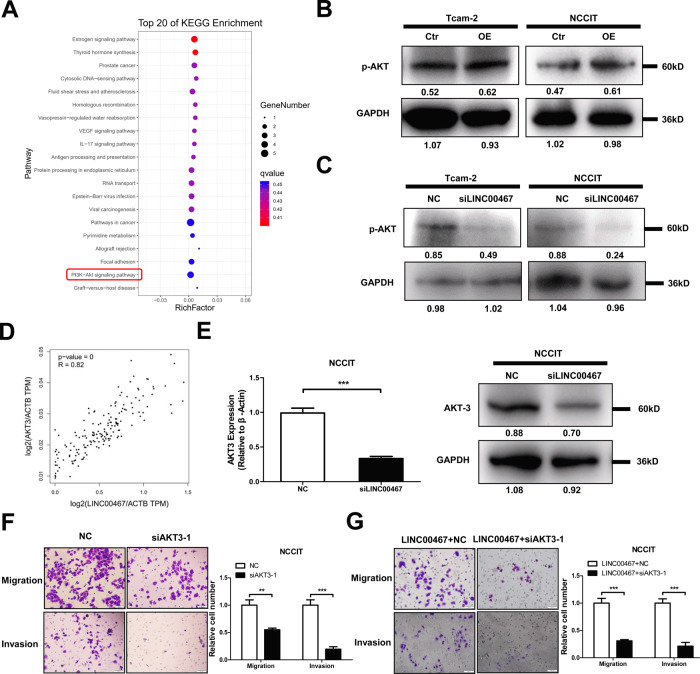
LINC00467 regulates AKT3-dependent AKT phosphorylation to promote the migration and invasion abilities of TGCT cells. **A** KEGG enrichment analysis of the genes co-expressed with LNC00467 in the Meturquoise module is shown in Fig. 4B. **B**, **C** Western blotting analysis of the change in the p-AKT level after the overexpression of LNC00467 (**B**) and LINC00467 silencing (**C**). **D** Data from the GEPIA database were used to determine the correlation between LINC00467 expression and AKT3 expression. **E** qRT-PCR and western blotting were used to determine the mRNA and protein levels of AKT3 after LINC00467 silencing. **F** Transwell cell migration and invasion assays were used to determine the effect of silencing on the migration and invasion abilities of TGCT cells. **G** Transwell cell migration and invasion assays were used to detect the effect of LINC00467 overexpression and simultaneous silencing of AKT3 on the migration and invasion abilities of TGCT cells. **P < 0.01; ***P < 0.001. All assays were performed in triplicate. Scale bar = 100 μm.

To explore the potential signaling pathways regulated by lncRNAs in the MMdarkGreen module, we analyzed the signaling pathways involved in the module. We found that the main signaling pathways that were enriched were “RNA transport,” “pathway in cancer,” and “PI3K-AKT signaling pathway” (Fig. 6A). Many studies have shown that the PI3K-AKT signaling pathway plays a key role in TGCT development and is involved in tumor progression through its regulation of processes, such as cell proliferation and migration [12, 13]. Our results showed that AKT phosphorylation increased after LINC00467 overexpression, whereas the silencing of LINC00467 decreased the level of AKT phosphorylation (Fig. 6B, C). Furthermore, GEPIA data mining showed that LINC00467 was significantly correlated with the transcriptional level of AKT3 (Fig. 6D). We have also carried out qRT-PCR to detect the positive correlation between LINC00467 and AKT3 mRNA expression in TGCT samples. There is indeed a certain positive correlation between the expression of LINC00467 and AKT3, but it may be because our sample size is too small, and there is no statistical significance (Supplemental Fig. 3A, *P* = 0.0554). Therefore, we speculated that LINC00467 might be involved in AKT phosphorylation by regulating AKT3 at the RNA level. To confirm the regulatory effect of LINC00467 on AKT3, we measured the mRNA and protein levels of AKT3 using qRT-PCR and western blotting analysis, respectively, after silencing LINC00467 in NCCIT cells. The results showed that at both mRNA level and protein level, AKT3 expression was significantly downregulated after LINC00467 silencing (Fig. 6E). These results suggest that LINC00467 can regulate the expression of AKT3. Since LINC00467 can affect the phosphorylation of AKT by regulating the expression of AKT3, we investigate whether AKT3 could also promote cell migration by affecting the phosphorylation of AKT. Subsequently, we examined the altered phosphorylation level of AKT by silencing AKT3 in cells and found a significant decrease after AKT3 silencing (Supplemental Fig. 3B, C). In terms of cell function, we found that rates of both migration and invasion decreased significantly after the silencing of AKT3 (Fig. 6F). Furthermore, we found a significant decrease in the rate of migration and invasion after AKT3 was silenced in conjunction with LINC00467 overexpression (Fig. 6G). This change suggests that AKT3 exerts a similar function to that of LINC00467 in promoting cell migration and invasion and that LINC00467 exerts its function by activating AKT3.

### LINC00467 is associated with immune cell infiltration

**Fig. 7:**
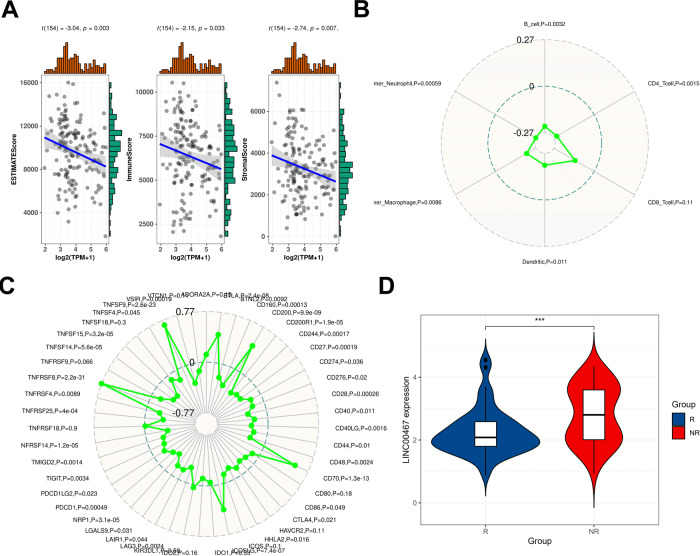
LINC00467 is negatively correlated with TGCT immune infiltration. **A** Correlation of LNC0040067 expression with estimate score, immune score, and stromal score. **B** Correlation between LNC00467 expression and the degree of immune cell infiltration. **C** Correlation between LNC00467 expression and immune checkpoint gene expression. **D** Expression of LNC00467 in samples from the response and non-response to immunotherapy groups. R: response, NS: non-response.

The tumor microenvironment is the cellular environment in which tumor cells are located and consists of an extracellular matrix, soluble molecules, and tumor stromal cells. In the tumor microenvironment, immune cells and stromal cells are the two main types of non-tumor components that have potential value for the clinical diagnosis and prognostic evaluation of tumors [14]. We downloaded TGCT data from the TGCA database and used ESTIMATE software to predict the correlation between LNC00467 and TGCT estimate score, immune score, and stromal score. The results showed that the expression was significantly negatively correlated with all three scores (Fig. 7A). We also found that the number of immune cells, such as neutrophils, macrophages, B cells, dendritic cells, and CD4+ T cells, was negatively correlated with the expression of LNC00467 (Fig. 7B).

Further analysis of immune checkpoint molecules revealed that LNC00467 was positively correlated with TNFSF4, TNFRSF9, CD70, and CD200 expression, and negatively correlated with CD274, CTLA4, and CD40 expression (Fig. 7C). Finally, we performed a predictive analysis to determine the response of how each sample to anti-PD-1 tumor immunotherapy. We also showed that the expression of LINC00467 was higher in the tumor immunotherapy non-responder group (Fig. 7D). Taken together, these results suggest that LNC00467 may exert an inhibitory effect on the infiltration and activation of immune cells and maybe a predictive target for anti-PD-1 immunotherapy.

## Discussion

LncRNAs can act as functional protein signals, guides, decoys, or scaffolds that can regulate gene expression at the epigenetic, transcriptional, post-transcriptional, and translational levels, thereby participating in tumor development [15]. For example, MALAT1 is a lncRNA that was one of the first to be associated with lung cancer metastasis, and it can exert pro-cancer effects through the activation of Wnt/β-catenin, EMT, and other signaling pathways [16]. Simultaneously, some lncRNAs are differentially expressed in various tumors and exert similar tumor-promoting or tumor-suppressing functions. HOTAIR is highly expressed in liver, lung, and pancreatic cancers and can significantly promote the metastasis of these tumors [5]. These results indicate that the role of lncRNAs in tumors is extensive and cannot be ignored.

In recent years, certain research teams have discovered that lncRNAs are involved in the pathophysiological processes of TGCT. For example, Sun Fei’s research group found that lncRNA NLC1-C can promote proliferation and inhibit apoptosis of testicular embryonic carcinoma cells through the adsorption of miR-320a and miR-383 [17]. A Norwegian research team found that lncRNA SPRY4-IT1 functions as a promoter of TGCT cell proliferation and invasion metastasis by promoting the phosphorylation of AKT [12]. These studies have just only begun to reveal information on TGCT-associated lncRNAs. In this study, we explored lncRNAs in TGCT tissues using high-throughput transcriptome sequencing and showed that many lncRNA transcripts, including new lncRNA transcripts can be found in TGCT tissues. Further analysis revealed that among the DElncRNAs between TGCT samples and paracancerous tissues, lncRNAs with a significantly downregulated expression accounted for >90% of all DElncRNAs. The higher proportion of downregulated lncRNAs suggests that many germ cell-specific lncRNAs may be dysregulated in testicular germ cell tumors.

LncRNAs include sense lncRNAs, antisense lncRNAs, bidirectional lncRNAs, intragenic lncRNAs, and intergenic lncRNAs. Among them, many antisense lncRNAs can bind to their homologous mRNAs through complementary base pairing to regulate them at the post-transcriptional level [18]. In this study, we identified signaling pathways associated with tumorigenesis, such as “cell adhesion,” “cytoskeleton organization,” and “cGMP-PKG signaling pathway,” which were significantly enriched by the enrichment and fractionation of antisense lncRNAs’ target genes. This finding further reflects the importance of the regulatory role played by antisense lncRNAs in TGCT. In addition, the bioinformatics enrichment analysis of *cis*- and *trans-*target genes of the lncRNAs showed that these target genes were also enriched in immune-related signal pathways, such as “nod-like receptor signaling pathway,” and “natural killer cell-mediated cytotoxicity.” Together these results indicate that lncRNAs can participate in the occurrence and development of TGCT through different regulation mechanisms.

The metastasis and recurrence of TGCT are one of the most intractable problems in a clinical setting. We found LINC00467 to be significantly associated with tumorigenesis, metastasis, and overall patient survival time. Simultaneously, subsequent data mining confirmed that LINC00467 may be a lncRNA that acts as a prognostic marker. LINC00467 was first reported to promote neuroblastoma survival and inhibit apoptosis [19]. Subsequent studies have demonstrated that LINC00467 can participate in the progression of various tumors [20, 21]. In the present study, we found that LINC00467 promoted the migration, invasion, and clone formation of TGCT cells, which is consistent with previous reports of other cancers and the first to be reported for TGCT. This finding suggests that LINC00467 may be a pan-tumorigenic gene. An in-depth investigation of the mechanism and clinical significance of this molecule will be of high significance for the diagnosis and treatment of various tumors. Although LINC00467 exerts similar functions in various tumors, its molecular mechanisms are not the same. It has been shown that LINC00467 promotes cell proliferation and migration in liver cancer by binding to IGF2BP3 to enhance the stability of its *cis*-target genes [22]. In esophageal cancer, LINC00467 can promote esophageal cancer cell proliferation by adsorbing miR-485-5p and reducing the inhibitory effect on the miRNA target gene, DPAGT1 [23]. Previously, our group found that LINC00467 can regulate the phosphorylation of AKT in non-small cell lung cancer by binding to AZGP1 to promote cancer cell migration and invasion [8]. Similarly, in this study, we found that LINC00467 promotes the migration and invasion of TGCT cells by regulating AKT phosphorylation. However, the mode of action by which LINC00467 regulates AKT phosphorylation in TGCT differs from that in lung cancer. We also found that LINC00467 is involved in regulating the RNA and protein level of AKT3, which affects the phosphorylation of total AKT. We suspect that LINC00467 may regulate the expression of AKT3 by affecting the stability or half-life of AKT3 RNA. However, this needs to be confirmed by a subsequent mRNA stabilization assay. Interestingly, AKT3 has been shown to promote germ cell tumor migration and invasion through the regulation of EMT [24]. These suggest that targeting the AKT3/AKT signal may be a potential therapeutic strategy that could counteract TGCT metastasis.

Malignant cells and complex types of non-malignant cells, including immune cells and stromal cells interact with each other to form a complex tumor microenvironment [25]. It has been shown that the degree of infiltration of immune cells, especially T cells, into the microenvironment of TGCT tumors is significantly correlated with the pathological grade and metastatic recurrence of TGCT [26]. In the present study, we found that LNC00467 expression was negatively correlated with immune score and stromal score and was significantly negatively correlated with T cells, macrophages, and other immune cells in the tumor microenvironment. Our results suggest that LNC00467 may be involved in the tumor immune process and the expression level of LNC00467 may serve as a potential predictor of response to tumor immunotherapy. However, this study was limited to in vitro experiments, and these findings need to be confirmed through subsequent in vivo experiments conducted on animals.

In summary, this is the first study to demonstrate that the pathophysiology of TGCT is associated with the dysregulation of lncRNAs. We also identified for the first time that LINC00467 performs important pro-migration and invasion functions in TGCT pathogenesis and may be a promising prognostic, immune marker, and therapeutic target.

## Materials and methods

### Testicular tumor and paraneoplastic tissue samples

We obtained 13 TGCT tissue samples (from 11 seminoma patients, abbreviated as SEM, and 2 non-seminoma patients with predominantly malignant embryonal carcinoma, abbreviated as non-SEM) and 4 paraneoplastic tissue samples (abbreviated as non-tumor) for RNA-Seq. We also collected 5 additional adjacent and 1 additional cancer tissue sample for qRT-PCR (9 non-tumors, 14 tumors in total). All samples were obtained from the Department of Urology, Affiliated Cancer Hospital, Xiangya School of Medicine, Central South University, with the approval of the Ethics Committee of Central South University. Informed consent was obtained from each patient who provided a sample.

### RNA extraction and quantitative real-time PCR (qRT-PCR)

Cellular and tissue RNA was extracted using the TRIzol method. In total, 1 µl of the total RNA was used to determine the OD and RNA concentration using a NanoDrop 1000 spectrophotometer. A 1% agarose gel was prepared, and 5 µl of total RNA was electrophoresed to determine RNA integrity. Reverse transcription PCR was performed using a Transcriptor First-strand cDNA synthesis Kit (Roche, USA), following the manufacturer’s instructions. The cDNA product was diluted fivefold with enzyme-free water, and then the LightCycler 480 SYBR Green I Master (Roche, USA) was operated as instructed by the manufacturer. Then, the target gene or lncRNA was standardized using the internal reference gene, β-actin. The relative expression of the target gene or lncRNA was calculated using the 2^−△△^ CT method to determine the differential expression of the target gene or lncRNA. The primer sequences were as follows: LINC00467 forward: 5’-TCGTCTTCAGGAAGCCAGAC-3’, reverse: 5’-TGGAAATCAAAAGGGTCAGC-3’; AKT3 forward: 5’-ACCGCACACGTTTCTATGGT-3’, reverse: 5’-CCCTCCACCAAGGCGTTTAT-3’; and β-actin forward: 5-CTGAGGATGCGAGGTTCTGCTTG-3, reverse: 5-GTCACCGGAGTCCATCACGAT-3.

### Transcriptome sequencing and bioinformatics analysis

We performed RNA sequencing through de-ribosomal strand-specific library sequencing by first removing rRNA from total RNA to retain the mRNAs and ncRNAs. Then, the enriched mRNAs and ncRNAs were fragmented into short fragments using a fragmentation buffer and reverse transcribed into cDNA using random primers. Second-strand cDNA was synthesized by adding DNA polymerase I, RNase H, dNTP (dUTP instead of dTTP), and a buffer. The cDNA fragments were purified using a QiaQuick PCR extraction kit to repair the ends, add poly(A), and ligate the Illumina sequencing joints. The second-strand cDNA was digested with UNG (uracil-N-glucanase). Agarose gel electrophoresis was performed to determine the size of the digested products. Then, the products were amplified using PCR and sequenced using an Illumina HiSeqTM 4000 platform (Gene Denovo, Guangzhou, China). The alignment of ribosomal RNA and reference genome was completed by Bowtie2 (2.2.8) and TopHat2 software, respectively. Cufflinks software was used to construct transcripts. The identification of new transcripts was carried out with Cuffcompare. CNCI (version 2) and CPC softwares were used to predict the protein-coding potential of novel transcripts. Quantification of transcripts abundance was performed by RSEM. The edgeR package was used to identify differentially expressed transcripts. A fold change ≥2 and a false discovery rate (FDR) <0.05 were identified as significantly differentially expressed. Gene Ontology (GO) enrichment analysis and pathway enrichment analysis were based on GO and KEGG databases, respectively. Antisense lncRNA target genes analysis was carried out by the software RNAplex. Genes in less than 100 kb up/downstream of a lncRNA were be identified as *cis*-target genes. Co-expressed genes not adjacent to lncRNAs, but the absolute correlation of more than 0.9 were considered as trans target genes. All the above target genes were then subjected to enrichment analysis of GO functions and KEGG pathways. Finally, we used the JASPAR tool to predict the transcription factors of lncRNAs and hierarchical clustering to analyze the relationship between samples.

### Weighted gene co-expression network (WGCNA) analysis

We extracted the expression data of 92 TGCT samples with complete clinical information from the TGCA data of the XENA database and then further extracted data onto the first 30,000 highly variable molecules (lncRNAs+mRNAs) for use in the WGCNA analysis. The WGCNA analysis was conducted using the R language software package WGCNA (v1.47) [27]. The modular partitioning of the WGCNA analysis was as follows: based on the variation of the average connectivity of genes with different power values, we selected 3 as the optimal power parameter for the subsequent co-expression network analysis (Supplementary Fig. 2A, C). The gene clustering tree (Supplementary Fig. 2B, D) was constructed based on the correlation between the expression of each gene. Then, the gene modules were divided according to the clustering relationship between genes (minimum number of genes in the module >50), and modules with similar expression patterns were merged based on the similarity of module features (similarity >0.8). The expression values of the first principal components of the gene modules in each sample were extracted from the analysis mentioned above. Finally, the correlation between the lncRNAs in the module and clinical phenotype was calculated based on Gene significance (GS) and Module Membership (MM) algorithms. The phenotypic data of our RNA sequencing data were generated by single-sample gene set enrichment analysis (ssGSEA). These phenotype-related gene sets were downloaded from the CancerSEA database. Modules with higher correlation and lower *P* values were selected to be used in the subsequent study.

### GEPIA database validation of DElncRNAs

We randomly selected six lncRNAs that were significantly upregulated and six lncRNAs that were significantly downregulated in the common differentially expressed lncRNAs of the three comparison groups (non-tumor vs SEM, non-tumor vs non-SEM, non-tumor vs SEM & non-SEM). The lncRNA symbols were entered into the GEPIA online tool. The TGCT dataset (tumor tissues) from TCGA and the GTEx dataset (normal testicular tissues) were selected for the differential analysis using cutoff values of |Log2FC|>1 and *P* < 0.01, and the images were exported.

### Cell culture, siRNA transfection, and vector construction

Associate Researcher Su-Ren Chen donated the cell line NCCIT (10% fetal bovine serum, FBS, RPMI1640). The other cell line, Tcam-2 (10% FBS, DMEM), was donated by Professor Yuxin Tang. Plasmids from the control and experimental groups were diluted with OPTI-MEM (Gibco, USA) following the manufacturer’s instructions if the Lipo3000 system (ThermoFisher Scientific), and a corresponding amount of Lipo3000 was dissolved in OPTI-MEM. Then, the reagents and samples were mixed and allowed to incubate for 15–20 m at room temperature. Finally, OPTI containing the plasmid and Lipo3000 complex was added into a Petri dish or plate for incubation in a humidified incubator at 37 °C. All siRNAs were designed and synthesized by Ribobio (Guangzhou, China). LINC00467 siRNA-1: GTCTTCAGGAAGCCAGACA; LINC00467 siRNA-2: GATGCTCTGTAAACCACAT; AKT3 siRNA-1: GGCAAGATGTATATGATAA; AKT3 siRNA-2: CCAGTGGACTACTGTTATA. The cDNA of LINC00467 was PCR-amplified and subcloned into the BamH1 and EcoRI sites of the pcDNA3.1(+) vector. The LINC00467 overexpression plasmid was designed and synthesized by Tsingke Biotechnology Co., Ltd (Beijing, China).

### Colony-formation assays

The cells transfected with negative control (NC) siRNA and LINC00467 siRNA (siLINC00467) for 24 h were digested, collected, and washed twice with pre-cooled 1×PBS. An appropriate amount of medium was added to adjust the cell concentration to 250 cells/ml. Then, 2 ml of the above-mentioned cell suspension was added into each well of a six-well plate. Then, the plate with cells was incubated in an incubator at 37 °C for 10 days, and the culture medium was renewed every 5 days. On the 10th day, the cells were washed twice with pre-cooled 1× PBS and stained with 0.1% crystalline violet staining solution for 15 m at room temperature.

### Transwell migration and invasion assays

Transwell assays were used to investigate the migration and invasion abilities of the tumor cells. The cells were seeded on the upper Transwell chambers with 8-μm pores (Corning, USA) at a density of 2 × 10^4^ cells per well with 200 µl of 2% FBS medium. Then, 800 μL of 15% FBS medium was added to the lower chamber of each Transwell chamber. Upper Transwell chambers with Matrigel were used for the invasion assays. After 48 h of incubation, each chamber was washed twice with PBS. Then, the cells that had invaded through the membranes were fixed using paraformaldehyde and were stained with crystal violet. Five random fields were photographed in each group, and the number of invaded cells were counted under a microscope.

### Western blotting assay

The cellular protein precipitates were extracted using a RIPA protein lysate containing a protease inhibitor Cocktail. Protein concentrations were determined using a BCA kit (ThermoFisher Scientific, USA). Then, the protein was subjected to SDS polyacrylamide electrophoresis and transferred onto a PVDF membrane. The PVDF membrane was blocked with 5% BSA at room temperature. Then, the PVDF membranes were incubated overnight at 4 °C with 5% skimmed milk and primary antibodies: AKT3 (Cell Signaling Technology), p-AKT (Cell Signaling Technology), and GAPDH (CoWin Biosciences). Then, the appropriate secondary antibody (CoWin Biosciences) was incubated with the PVDF membranes based on the genetic origin of the primary antibody. A gel imaging system was used to scan the protein bands using ECL reagents, and GAPDH was used as an internal reference.

### Immunoinfiltration analysis

The correlation analysis between immune scores, immune cell infiltration, immune checkpoint molecules, and LINC00467 expression was conducted based on TCGA TGCT cohort data by R software. Immunotherapy response prediction was also conducted based on the TCGA TGCT cohort data, and the analysis was performed using the ImmuCellAI online tool (http://bioinfo.life.hust.edu.cn/ImmuCellAI#!/analysis) with default parameters [28].

### Statistical analysis

All data were statistically analyzed using GraphPad Prism 5 software, and Student’s *t* test was used to calculate the significance of the difference between the two groups. Significant differences between multiple data sets were calculated using one-way ANOVA. Overall survival and disease-free survival were calculated using a log-rank test.

## Supplementary information

Supplemental Figure 1

Supplemental Figure 2

Supplemental Figure 3

## GLOSSARY

lncRNAs: long noncoding RNAs
TGCT: testicular germ cell tumor
WGCNA: weighted gene co-expression network analysis
qRT-PCR: quantitative real-time PCR
SEM: seminoma
non-SEM: non-seminoma
OS: overall survival
GS: gene significance
MM: module membership
ssGSEA: single-sample gene set enrichment analysis
