# Supplementary figures and images for: Integrated analysis of high-throughput sequencing data reveals the key role of LINC00467 in the invasion and metastasis of testicular germ cell tumors
Source: Cell Death Discov. 2021 Aug 6;7:206. doi: 10.1038/s41420-021-00588-9 (PMC8346510; doi:10.1038/s41420-021-00588-9)

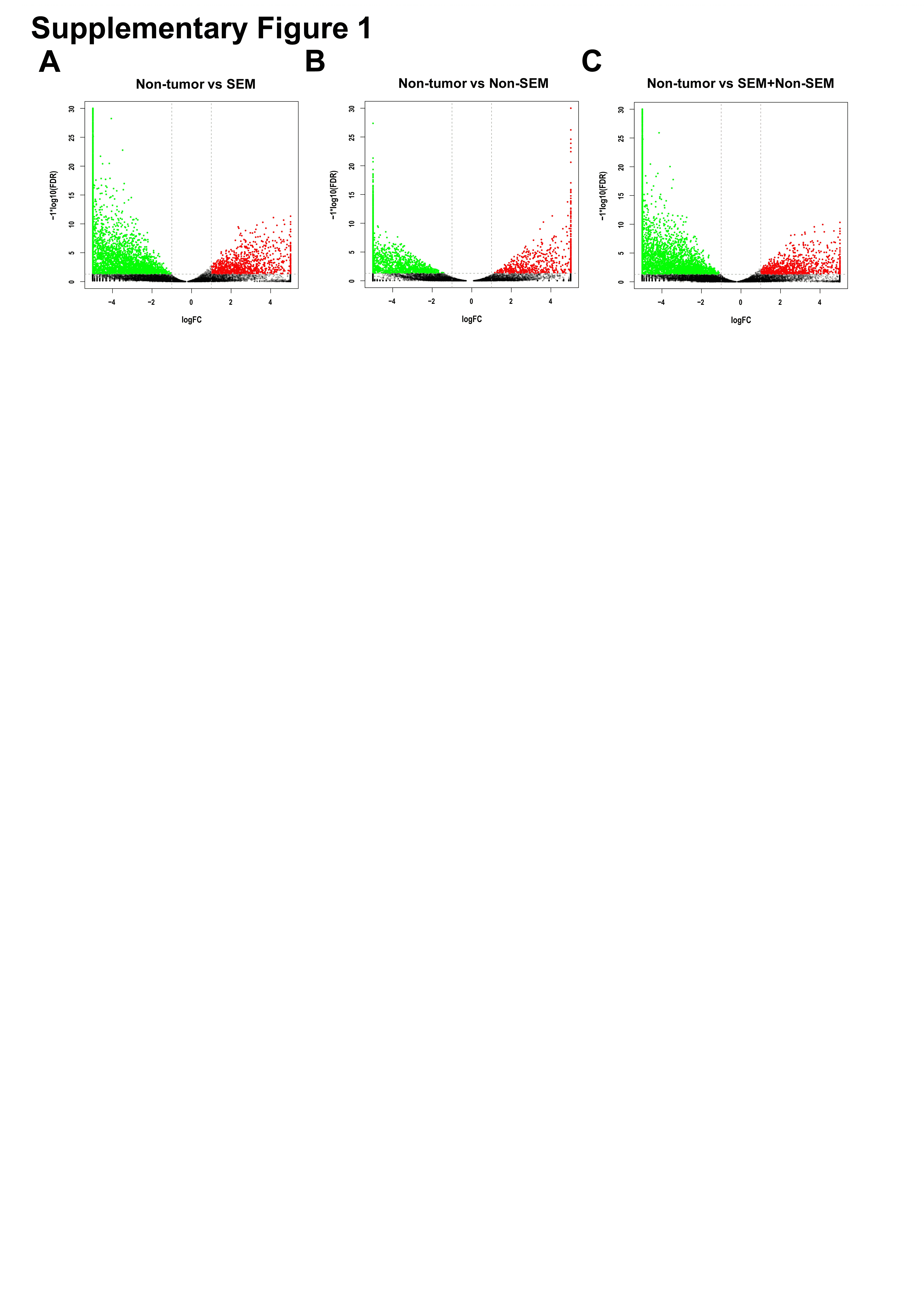

Supplement: Supplementary file 1 — Supplemental Figure 1 [file 41420_2021_588_MOESM1_ESM.png]

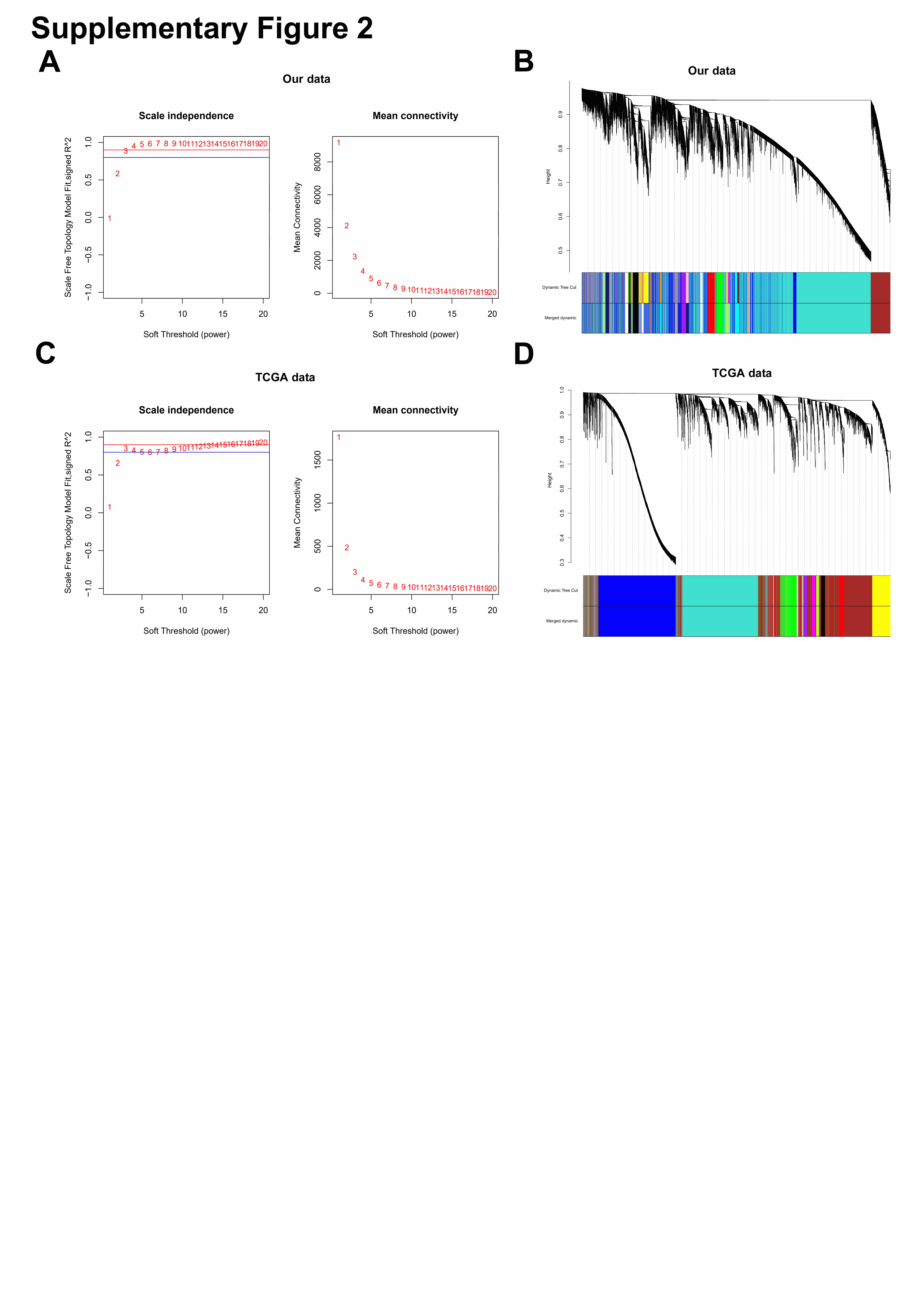

Supplement: Supplementary file 2 — Supplemental Figure 2 [file 41420_2021_588_MOESM2_ESM.png]

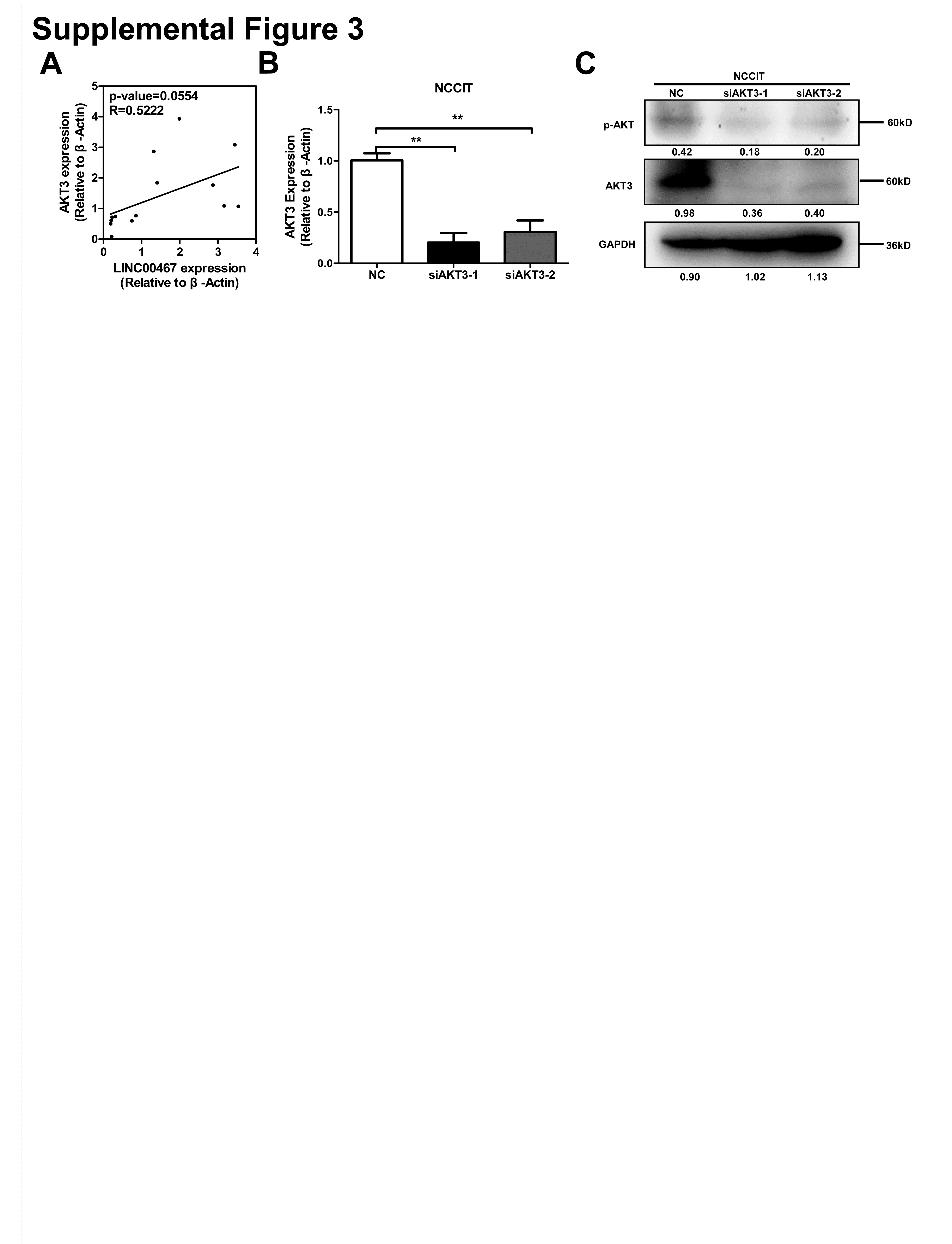

Supplement: Supplementary file 3 — Supplemental Figure 3 [file 41420_2021_588_MOESM3_ESM.png]
